# A Comparison between Use of Spray and Freeze Drying Techniques for Preparation of Solid Self-Microemulsifying Formulation of Valsartan and *In Vitro* and *In Vivo* Evaluation

**DOI:** 10.1155/2013/909045

**Published:** 2013-07-18

**Authors:** Sanjay Kumar Singh, Parameswara Rao Vuddanda, Sanjay Singh, Anand Kumar Srivastava

**Affiliations:** Department of Pharmaceutics, Indian Institute of Technology (Banaras Hindu University), Varanasi 221005, India

## Abstract

The objective of the present study was to develop self micro emulsifying formulation (SMEF) of valsartan to improve its oral bioavailability. The formulations were screened on the basis of solubility, stability, emulsification efficiency, particle size and zeta potential. The optimized liquid SMEF contains valsartan (20% w/w), Capmul MCM C8 (16% w/w), Tween 80 (42.66% w/w) and PEG 400 (21.33% w/w) as drug, oil, surfactant and co-surfactant, respectively. Further, Liquid SMEF was adsorbed on Aerosol 200 by spray and freeze drying methods in the ratio of 2 : 1 and transformed into free flowing powder. Both the optimized liquid and solid SMEF had the particle size <200 nm with rapid reconstitution properties. Both drying methods are equally capable for producing stable solid SMEF and immediate release of drug in *in vitro* and *in vivo* conditions. However, the solid SMEF produced by spray drying method showed high flowability and compressibility. The solid state characterization employing the FTIR, DSC and XRD studies indicated insignificant interaction of drug with lipid and adsorbed excipient. The relative bioavailability of solid SMEF was approximately 1.5 to 3.0 folds higher than marketed formulation and pure drug. Thus, the developed solid SMEF illustrates an alternative delivery of valsartan as compared to existing formulations with improved bioavailability.

## 1. Introduction

The literature survey suggests that about 40% new drug candidates have low aqueous solubility, which leads to poor bioavailability, high inter-/intrasubject variability, and lack of the dose proportionality [1, 2]. In biopharmaceutical classification system (BCS), such type of drug comes under class II which shows low solubility and high permeability [3]. The bioavailability of these drugs mostly depends upon the dissolution. Several formulation strategies such as utilization of water soluble carriers, surfactants, lipids, polymeric conjugates, and solid dispersion have been adopted by the researchers for enhancement of solubility and dissolution properties of BCS class II drugs [4–6].

Since past decade much attention has been focused on lipid drug delivery system such as lipid solution and suspension [7] emulsion [8] solid dispersion [9] self-microemulsifying formulation (SMEF) [10], liposomes [11]. The self-microemulsifying formulation (SMEF) is one of the novel approaches for delivery of low aqueous soluble lipophilic drugs [12–15]. SMEF is an isotropic mixture of oil, surfactant, and cosurfactant which can be converted to emulsion with aqueous media under gentle agitation. It can be prepared either in liquid form or encapsulated in hard or soft gelatin capsule. Nevertheless, it has some drawbacks also such as instability, leakage, precipitation of drug, and ageing of shells of the capsules [16]. To solve these above problems, the researchers have successfully developed solid SMEF using solid carriers and demonstrated their usefulness in dissolution and bioavailability. Recently the spray drying method for solidification of SMEF has been reported using different adsorbent materials for enhancement of solubility and bioavailability of coenzyme Q10, nitrendipine, and nimodipine [10, 17, 18]. Several other different techniques have also been reported for the solidification of liquid SMEF [19, 20]. However, it is necessary to evaluate the behavior of solid SMEF carriers for better understanding of their ability in *in vivo* conditions. 

Valsartan is an angiotensin II receptor blocker, antihypertensive drug [21, 22]. It is a BCS class II drug and its low aqueous solubility contributes to poor bioavailability (23%) [23, 24]. Few approaches have been made for the improvement of solubility of valsartan such as use of hydroxypropyl-*β*-cyclodextrin and methyl-*β*-cyclodextrin valsartan dispersions [25, 26], but we could not predict *in vivo* performance of those dispersions. Apart from this, a liquid self-emulsifying formulation has been reported for valsartan, but it still has the inherent problem of liquid dosage form as discussed above [27]. 

The objective of the present study was to develop solid self-microemulsifying formulation (S-SMEF) of valsartan by spray drying (SD) and freeze drying (FD) methods and evaluate them with respect to liquid SMEF as well as marketed formulation. The formulations were evaluated for flow properties, redispersibility capacity, particles size, change in the physicochemical properties of drug during the process, dissolution profile of valsartan from its different solid SMEF, and *in vivo* bioavailability. 

## 2. Materials and Methods

### 2.1. Materials

Valsartan and Aerosil 200 were obtained from Ranbaxy, India, as gift sample. The Labrafac Lipophile (derivative of caprylic/capric triglyceride), Labrafil M 2125 CS (polyoxyethylated glycolysed glycerides), and Labrasol (caprylocaproyl polyoxyl-8 glycerides) were purchased from Gattefosse, India. Capmul MCM C8 (mono-/diglycerides of caprylic acid) and Captex 355 (caprylic/capric triglyceride derivative) were obtained as gift sample from Abitec, UK, Miglyol 812 (caprylic/capric triglyceride) was gifted by Sasol Germany Gmbh. Tween 80 (polyoxyethylene sorbitan monooleate), Tween 20 (polyoxyethylene sorbitan monolaurate), PEG 400, PEG 600, propylene glycol, and glycerol were purchased from S.D. fine-chem Ltd, India. All other chemicals used in the research work were of analytical grade and used as obtained. 

### 2.2. Methods

#### 2.2.1. Screening of Excipients

The solubility study was performed to select the suitable oil (O), surfactant (S), and cosurfactant (Co-S) that possesses high solubilizing capacity for valsartan. The various oils such as medium-chain di- and triglycerides (Labrafac Lipophile, Labrafil M 2125 CS, Miglyol 812, Captex 355, and Capmul MCM C8) and long-chain triglycerides (soybean, sunflower and castor oil), surfactants (Tween 80, Tween 20, and Labrasol), and co-surfactants (PEG 400, PEG 600, propylene glycol, and glycerol) were selected and their solubility was determined by shaking flask method. The excess amount of drug was placed in 5.0 mL screw cap glass bottle having 2.0 mL of each oil, surfactant, and co-surfactant. The glass bottle was placed on magnetic stirrer (IKA RCT Basic, India) and stirred for 24 hour at 40°C. The saturated lipid solution was further centrifuged at 15000 rpm for 15 minutes to remove insoluble drug. The 100 *μ*L of supernatant was withdrawn and diluted appropriately with methanol. The drug concentration in filtrate was determined by UV-spectrophotometer (U-1800 Hitachi, Japan) at *λ*_max⁡_ 250 nm [28, 29].

#### 2.2.2. Preparation of Liquid SMEF

A series of formulations were prepared by varying the proportion of surfactant, cosurfactant, and oil phase. The drug valsartan was dissolved in the mixture of oil, surfactant, and cosurfactant at ambient room temperature. The composition of formulations is given in Table 1. The drug was initially dissolved in oil phase followed by addition of mixture of S and Co-S in a glass vial. The final mixture was vortexed until a clear solution was obtained.

#### 2.2.3. Preparation of Solid SMEF

The optimized liquid self-microemulsifying formulation (L-SMEF) was transformed into free flowing granules using Aerosil 200 colloidal porous carriers as adsorbent. Two different techniques (spray drying and freeze drying) were used for the adsorption of L-SMEF to form solid SMEF (S-SMEF). The L-SMEF and Aerosil 200 were taken in two different ratio 1 : 1 and 2 : 1 w/w to optimize the drug loading on colloidal silica with two methods (data not shown). On the basis of higher drug load and flow properties, the ratios of 2 : 1 w/w was selected for further studies. The solid self-microemulsifying spray dried (S-SMSD) and solid self-microemulsifying freeze dried (S-SMFD) formulations were further evaluated with optimized liquid SMEF.

*Spray Drying Method*. Twenty grams of L-SMEF was added to 500 mL distilled water and stirred (100 rpm) for 10 min, to form homogeneous fine emulsion. Further, 10 g of Aerosil 200 was added to the prepared emulsion and mixed by stirring at 100 rpm for 10 min. The above Aerosil suspension was spray dried by Jay LSD-48 Mini Spray dryer (Jay Instrument & System Pvt. Ltd., Mumbai) at the following specification: inlet temperature, 105°C; outlet temperature, 70°C; aspiration, 85%; drying air flow, 500 NL/h; and feeding rate of the emulsion, 2.5 mL/min.

*Freeze Drying (Lyophilization) Method*. The microemulsion containing solid adsorbent was prepared as described in spray drying method. The Aerosil suspension thus obtained was freeze dried to remove water by sublimation methods. No lyoprotectant for freeze drying process was used as Aerosil itself had inherent properties of lyoprotectant [30]. The lyophilizer (Decibel Digital Technology, India) was operated at condenser temperature −60°C and pressure below 15 Pascal.

#### 2.2.4. Evaluation of Liquid and Solid SMEF

*Emulsification Efficiency. *One milliliter of L-SMEF was dispersed in 100 mL distilled water. Further, the previous suspension was kept for stirring at 100 rpm for 30 min at 25°C on a magnetic stirrer. The same procedures were followed for determining emulsification efficiency of S-SMSD and S-SMFD by taking 50 g of solid SMEF. The formulations were visually observed using the following grading system [31, 32]:

- denotes a rapidly forming, slightly less clear emulsion which has a bluish-white appearance;
- denotes a bright milky white emulsion that formed within 2 min;
- denotes a dull, grayish-white emulsion with slightly oily appearance, slow emulsification (more than 2 min);
- denotes a formulation which exhibited either poor or minimal emulsification with large oil droplets present on the surface.

*Droplet Size, Polydispersity Index, and Zeta Potential Analysis*. Separately 1.0 mL of L-SMEF and 10 mg of S-SMEF were diluted with 100 mL distilled water with constant stirring at 100 rpm for 15 minutes. The droplet size, polydispersity index, and zeta potential of resultant microemulsion were determined by particle size analyzer (Delsa Nano C, Beckman Coulter, UK) based on dynamic light scattering at 165° at 25°C. All studies were performed in triplicate and their mean values were reported [33].

*Freeze Thawing.* Freeze thawing was employed to evaluate the thermal stability of liquid SMEF. The formulations were subjected to 3 to 4 freeze-thaw cycles, which included freezing at −4°C for 24 hours followed by thawing at 40°C for 24 hours. The formulations were then observed for phase separation [12].

*Drug Content*. The prepared S-SMSD, S-SMFD, and liquid SMEF (F9) equivalent to 40 mg of valsartan were dissolved in sufficient quantity of ethanol and vortexed for 2.0 h, followed by centrifugation at 2000 rpm for 15 min. The supernatant was filtered through 0.45 *μ*m Whatman filter paper (USA) and further diluted with distilled water. The drug content was determined by UV spectrophotometer at *λ*_max⁡_ 250 nm.

#### 2.2.5. Powder Properties of Solid SMEF

S-SMSD and S-SMFD were evaluated for flowability and compressibility parameters. The flowability of the powder formulations was determined by the angle of repose. Angle of repose is defined as the maximum angle possible between the surface of a pile of the powder and the horizontal surface. It is a direct indication of potential flowability. The lower the angle of repose, the better the flow property and vice versa. The compressibility of the powder is expressed as Carr's index and Hausner ratio. It is derived from tapped density and fluff density. The tapped density was measured after 100 tapping of powder in measuring cylinder [34, 35]. Hausner ratio less than 1.25 (equivalent to 20% Carr's or angle of repose less than 30°) indicates good flowability and compressibility of the material:

(1) $$\begin{array}{c} \text{Carr's}\;\text{index}=\frac{\text{tapped}\;\text{density}-\text{fluff}\;\text{density}}{\text{tapped}\;\text{density}}\times 100\\ \text{Hausner}\;\text{ratio}=\frac{\text{tapped}\;\text{density}}{\text{fluff}\;\text{density}}. \end{array}$$

#### 2.2.6. Morphological Analysis of Solid SMEF

The surface characterization of the valsartan, Aerosil 200, S-SMSD, and S-SMFD was investigated by a high-resolution field emission scanning electron microscopy (SEM, FESEM-Carl Zeiss, Supra 40) operated at an accelerating voltage 3.0 kV. Prior to imaging, samples were sputter coated for 50 s with platinum using a JEOL JFC-1200 fine coater to make the conducting specimens. 

#### 2.2.7. Solid-State Characterization of Solid SMEF

*Differential Scanning Calorimetry (DSC). *The physical state of valsartan, Aerosil 200, S-SMSD, and S-SMFD was characterized by the differential scanning calorimetry (Diamond DSC, Perkin Elmer, USA). DSC was performed to evaluate any changes in melting enthalpy, glass transition temperature, and percentage of crystallinity in respect to any interactions with excipient. About 3.0 mg of sample was placed in standard aluminum pans, and dry nitrogen was used as effluent gas. All samples were scanned in the range of 20 to 200°C at a temperature increment speed of 10°C/min.

*X-Ray Powder Diffraction (XRD). *X-ray powder scattering measurements were carried out to check the crystallinity of drug in pure and solid SMEF. The studies were done using 18-kW rotating anode (Cu-target) based Rigaku powder diffractometer (Tokyo, Japan) operating in the Bragg-Brentano geometry and fitted with a curved crystal graphite monochromator in the diffracted beam. The solids were scanned over a range of 2*θ* angles from 10° to 40°, at an angular speed of 2° (2*θ*)/min, and a sampling interval of 0.02°.

#### 2.2.8. Drug-Excipient Interaction Study

The I.R. spectra of valsartan, liquid, and solid SMEF were recorded by Fourier transform infrared spectroscopy (FTIR Shimadzu, Japan). Sample preparation involved mixing the sample with potassium bromide (KBr) in 1 : 50 ratio, triturated in glass mortar, pelletized, and finally placed in sample holder. The spectrum was scanned over the frequency range of 4000–400 cm^−1^. 

#### 2.2.9. *In Vitro *Dissolution Studies

*In vitro* dissolution study of S-SMEF was performed by using USP Dissolution Apparatus II. Solid SMEF (S-SMSD and S-SMFD) formulations equivalent to 40 mg valsartan were filled into hard gelatin capsules and put into the dissolution vessels. The capsule was kept at bottom with the help of sinker made up of stainless steel. The dissolution vessel was fitted with 900 mL dissolution media (pH 1.2 and 6.8 buffer) and kept at 37 ± 0.5°C with a rotating speed of 50 rpm. The aliquot of 5.0 mL was withdrawn at 10, 20, 30, 40, 50, and 60 min and filtered through 0.45 *μ*m Whatman membrane filter (USA). The volume withdrawn was replaced each time by fresh dissolution media. For calculation of cumulative % drug release, The correction factor for drug loses during sampling was calculated as per the following formula:

(2) $$\begin{array}{c} C_{c}=C_{i}+\frac{V_{s}}{V_{t}}\times \sum_{i=1}^{n-1}C_{i}, \end{array}$$

where *C*_*c*_ is the corrected concentration, *C*_*i*_ is the uncorrected concentration, *V*_*s*_ is the volume of sample withdrawn, and *V*_*t*_ is the total volume of dissolution medium [36]. The dissolution profile of solid SMEF was compared with liquid SMEF, pure drug, and marketed formulation (Valzaar 40 mg, Torrent Pharma, India). The amount of valsartan released in dissolution medium was determined by UV-spectrophotometry method at *λ*_max⁡_ 250 nm. The dissolution study was performed in triplicates [13].

#### 2.2.10. Accelerated Stability Study

The accelerated stability study was carried out according to the International Conference on Harmonization (ICH) guidelines (Q1C and Q1A (R2)). Sealed vials of freshly prepared spray and freeze dried SMEF were placed in stability chamber maintained at 40°C ± 2°C/75%  RH ± 5%  RH. The liquid SMEF and solid SMEF subjected to stability tests were analyzed over 3-month period for physical appearance, reconstituted globule size, and total drug content.

#### 2.2.11. *In Vivo* Pharmacokinetic Study

The animal study was approved and performed in accordance with the guidelines of the Central Animal Ethics Committee of the University, Banaras Hindu University, Varanasi (Ref. no. Dean/11-12/CAEC/324). The prepared formulations (L-SMEF (F9), S-SMSD, and S-SMFD) were compared with valsartan suspension and marketed formulation. Male Sprague-Dawley (SD) rats (250–280 g) were used in this study. The rats were fasted for 12 h prior to experiment, fed at 4 h later dosing, and water was accessed *ad libitum* throughout the study period. Thirty rats were divided into five groups of six rats each. Each group was administered with pure valsartan, marketed formulation, liquid SMEF (F9), S-SMSD, and S-SMFD each at the dose of 5.0 mg/kg. The blood samples (500 *μ*L) were collected at predetermined time intervals through retro-orbital puncture and plasma was separated by centrifuging blood sample at 5,000 rpm for 15 min. Plasma sample was stored at −20°C until further analysis. 

The drug in plasma samples was quantified by HPLC methods as reported by Li et al. [37]. Plasma (200 *μ*L) was mixed with 300 *μ*L of methanol. Then, 1.5 mL of t-butyl methyl ether was added, vortexed for 2 min, and centrifuged at 5000 rpm for 10 min. The supernatant layer was separated and evaporated at 40°C. The residue was further reconstituted by 200 *μ*L of mobile phase. The resulting solution (20 *μ*L) was analyzed by Waters HPLC 515 having Rheodyne 7725i injector fitted with 20 *μ*L loop and equipped with photodiode array (PDA) 2998 detector (Waters, USA) using a Waters column, C18 spherisorb 5.0 *μ*m ODS2 4.6 mm × 250 mm column. The mobile phase consisted of phosphate buffer (pH 3.0) and acetonitrile (40/60) at a flow of 1.0 mL/min. Data was further processed by Empower Pro 2 software at *λ*_max⁡_ 250 nm. 

The area under the drug concentration-time curve (AUC), peak plasma concentration (*C*_max⁡_) and its time (*t*_max⁡_), elimination constant (Kel), and half-life (*t*_1/2_) were calculated using a noncompartmental analysis (WinNonlin Software, version 5.3). Student's *t*-test was performed to evaluate the significance difference between prepared formulations with pure drug and marketed formulation. The values were reported as mean ± SD with *P* value (<0.05). 

## 3. Results and Discussion

### 3.1. Screening of Excipients

**Figure 1:**
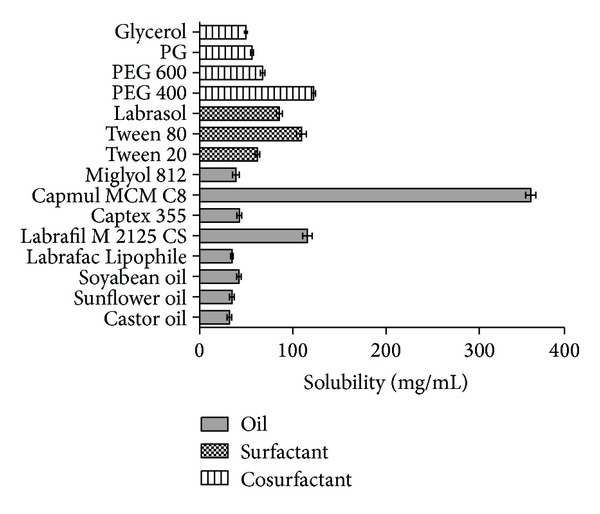
Valsartan solubility in various oils, surfactants, and co-surfactants.

The solubility of valsartan among selected oils was highest in Capmul MCM C8 and it was 355.3 ± 5.5 mg/mL, whereas in surfactant and cosurfactant the maximum solubility of valsartan was found as 109.0 ± 2.6 mg/mL and 122.3 ± 2.0 mg/mL in Tween 80 and PEG 400, respectively (Figure 1). Valsartan had higher solubility in medium-chain diglyceride and triglyceride as compared to long-chain triglyceride. It might be due to low molecular weight (435.5) and moderate hydrophobicity (log *P* = 1.5) of valsartan which favors the high solubility in capmul MCM C8 containing C8–C10 carbon chain. The solubility determination of drug in various lipids is very essential because the higher solubility of the drug in oil phase and other components is the desired element for SMEF to keep volume of the formulation as minimum as possible to deliver the therapeutic dose of the drug in an encapsulated form. The higher solubility of drug in lipids is also helpful in avoiding precipitation of the drug on dilution in the gut lumen *in vivo* [38]. Further, the S and Co-S with higher solubility of valsartan were selected to provide the additive effect into the drug loading. The presence of cosurfactant is mainly accountable for the rapid penetration of the aqueous phase into the lipid and instant formation of microemulsion. Moreover, the selected lipids were checked for miscibility to each other. The blank L-SMEFs consisting of Capmul MCM C8, Tween 80, and PEG 400 were completely miscible to each other and no separation was observed for two weeks (data was not shown). 

### 3.2. Preparation of Liquid SMEF

Mixtures of Tween 80 and PEG 400 were prepared in the ratios of 1 : 1, 2 : 1, and 3 : 1 w/w. Total fifteen batches (F1–F15) were prepared by varying ratio of S/Co-S phase (40–72% w/w) and oil phase (8–40% w/w). The weight of the drug ratio was kept constant (20% w/w) with respect to total amount of the excipients such as oil, surfactant, and cosurfactant (80% w/w) in all batches. The efficiency of emulsification was good when the S/Co-S concentration was more than 70% w/w of the SMEF formulation. Moreover, increasing the ratio of S/Co-S in the SMEF formulation resulted in more fine formulation and it was observed in all three S/Co-S ratios. However, all formulations having S/Co-S ratio 2 : 1 were not stable in freeze thaw cycle, but fine and instance microemulsion formation was observed as compared to S/Co-S ratios 1 : 1 and 3 : 1. These findings indicated that ratio 2 : 1 had optimum concentration of surfactant and cosurfactant for solubilization and formation of fine microemulsion. 

### 3.3. Preparation of Solid-SMEF

**Figure 2:**
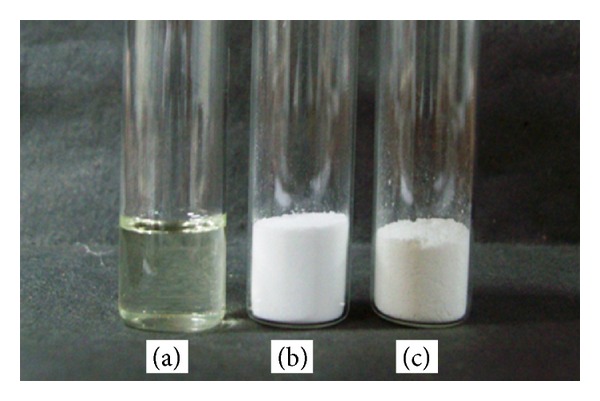
Photograph showing (a) L-SMEF (F9), (b) S-SMSD, and (c) S-SMFD.

Based on the optimization parameter (as described in Sections 3.4.1 and 3.4.2) the batch F9 was selected for solidification process by using Aerosil 200 as adsorbent material. The spray and freeze dried techniques were used for solidification of the liquid SMEF. The yield in spray and freeze drying was 78% and 100%, respectively. The low yield in spray dryer was due to loss of very fine particles as these particles could not settle on the cyclone collector chamber, whereas in freeze dryer no loss of particles and complete recovery of formulation were found (Figure 2). 

### 3.4. Evaluation of Liquid and Solid SMEF 

#### 3.4.1. Efficiency of Self-Emulsification and Reconstitution Properties

The results of the self emulsification efficiency of L-SMEF are mentioned in Table 2. The self-emulsifying formulations F5, F9, F10, and F15 exhibited immediate formation of emulsion with slight appearance, and it did not show residual oil on the surface of emulsion. These batches were considered better formulations on the basis of emulsification and reconstitution properties. The batches F4, F8, and F14 showed self-emulsion with milky appearance but failed in instant microemulsion formation within 2 min. The batches F1, F11, and F12 were poorly emulsified, and phase separation occurred within 30 min. This poor emulsification was due to low concentration of mixture of S and Co-S. Due to this reason, the water molecules were not able to penetrate the oil phase easily and produce large oil globule on water surface. The batches F2, F3, F6, F7, and F13 were of dull, grayish white with a slightly oily appearance, and emulsification time was longer than 2 minutes. 

**Figure 3:**
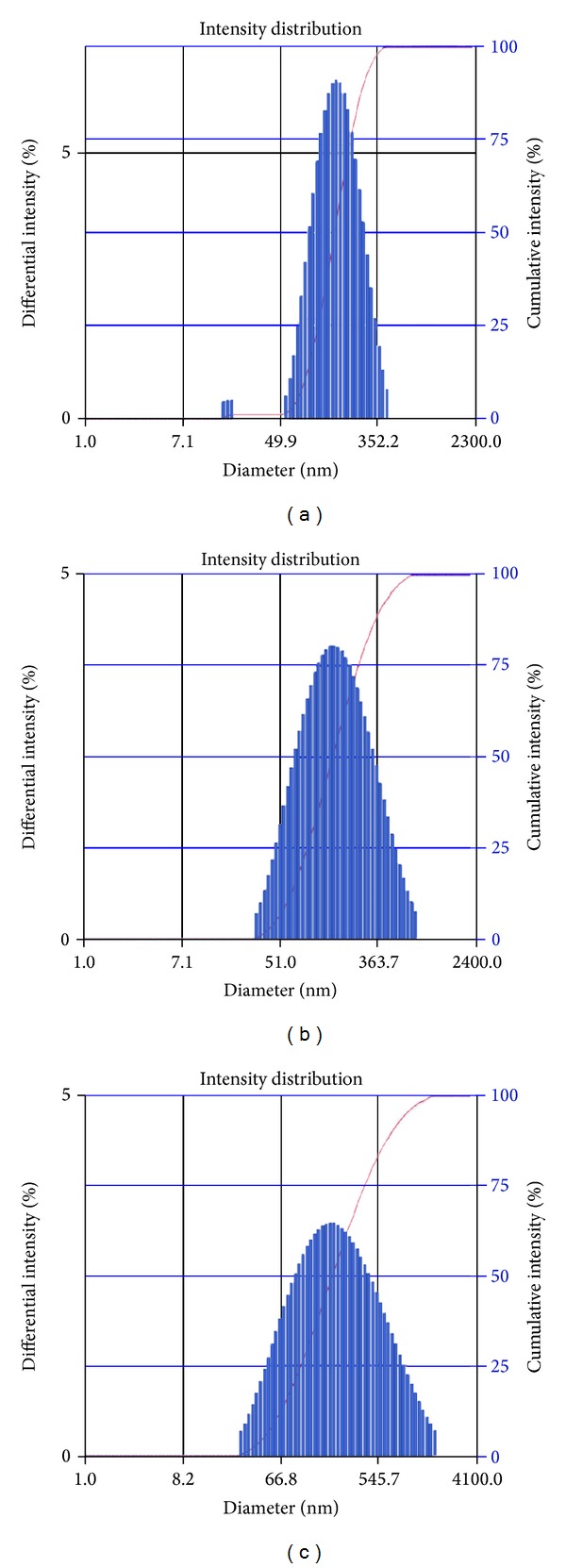
Globule size distribution in (a) L-SMEF, F9, (b) S-SMSD, and (c) S-SMFD.

The solid SMEF (S-SMSD and S-SMFD) prepared by spray drying and freeze drying methods exhibited a rapidly forming, slightly less clear emulsion which had a bluish-white appearance after reconstitution. The formation of rapid microemulsion after reconstitution of solid SMEF indicates the retention of self-emulsifying properties in solid form (Figures 3(b) and 3(c)). 

#### 3.4.2. Droplet Size, Zeta Potential, and Freeze Thaw Studies

Results of droplet size and zeta potential are depicted in Table 2. The prepared batches had globule size in the range of 105 to 1238 nm. The globule size of F10 batch was 105 nm followed by the batch F9 having globule size of 127 nm. In batch F10, the globule size increased with time and precipitation of drug occurred during freeze thaw study. It may be attributed to the loss of solubilization properties of excipient which leads to precipitation and phase separation. The zeta potential of all liquid SMEF formulations was found to be near to zero justifying the nonionic nature of surfactant and co-surfactant. The batch F9 was selected as best liquid SMEF formulation composition based on the low particle (127 nm), high zeta potential (4.6 mV), freeze thaw stability, and high emulsification efficiency. 

The average droplet size of both S-SMSD and S-SMFD was less than 200 nm. The larger particle size and wide size distribution were observed in S-SMFD when compared with liquid SMEF (F9); however, this difference was not statistically significant (*P* < 0.05). From these results, adsorption of liquid SMEF on Aerosil 200 by spray drying and freeze drying did not seem to have a remarkable effect on droplet size, and it retained the self-emulsification performance of the liquid SMEF (Table 3). 

#### 3.4.3. Drug Content

The drug content was determined in liquid SMEF (F9), S-SMSD, and S-SMFD, and data is presented in Table 3. The drug content was 99.32% and 99.14% for liquid SMEF and S-SMFD, respectively, whereas it was 97.59% in S-SMSD. The lower drug content in S-SMSD was probably due to the loss in removal of nonencapsulated free drug with the exhausted gas [39]. 

### 3.5. Powder Properties of Solid SMEF

The flow properties of both solid SMEFs are compared, and the finding is presented in Table 4. The angle of repose of S-SMSD exhibited lower value (30°) as compared to S-SMFD (38°). The presence of round and uniform sphere may be responsible for lower value of angle of repose in S-SMSD, and it was also confirmed by SEM image. The higher value of tapped density (0.606 ± 0.042 mg/cc) and fluff density (0.488 ± 0.017 mg/cc) and lower value of compressibility index (19.5 ± 1.3) and hausner ratio (1.242 ± 0.21) for S-SMSD indicate weak interparticle interaction and higher flow rate as compared to S-SMFD. The above powder flow properties indicated that the solid SMEF can be used in preparation of tablet, pellets, and capsule dosage form. 

### 3.6. Morphological Analysis of Solid SMEF

**Figure 4:**
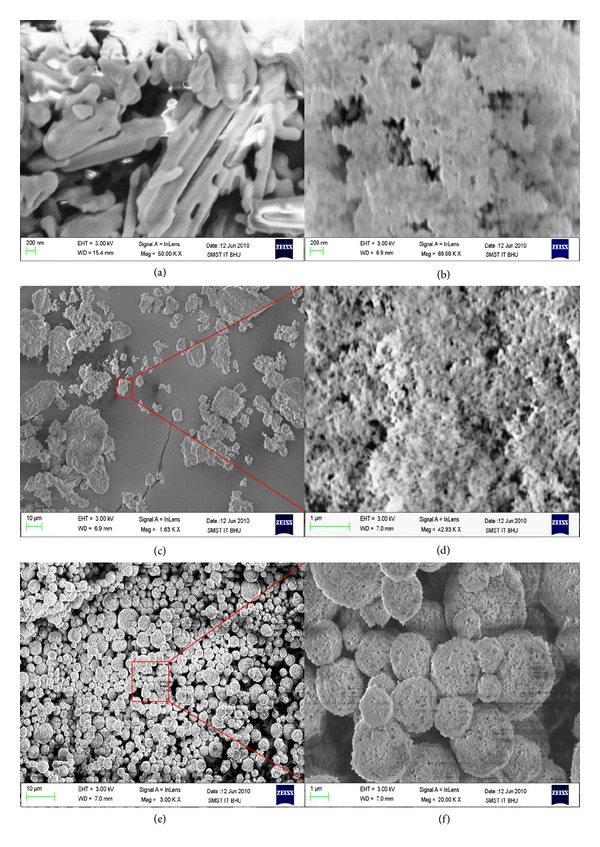
Scanning electron microscope graph of (a) valsartan; (b) Aerosil 200; (c) S-SMFD, 1.6 KX; (d) S-SMFD, 42.93 KX; (e) S-SMSD, 3 KX; and (f) S-SMSD, 20 KX.

The morphology of valsartan particles, Aerosil-200, and both solid SMEFs was analyzed by SEM. The valsartan showed crystal, needle-shape irregular particles (Figure 4(a)), whereas Aerosil 200 had irregular mass with different size and shape, but surface of the particles was found to be of porous nature, which clearly justified its use for adsorption of liquid emulsifying formulation (Figure 4(b)). The S-SMFD was irregular and aggregate particles of Aerosil. It was due to the freeze drying process in which particles were not able to segregate. The S-SMSD exhibited spherical-shaped, porous particles size (Figures 4(e) and 4(f)). The magnified micrograph of S-SMFD and S-SMSD formulation revealed the presence of porous surface responsible for higher solubility and instant formation of microemulsion (Figures 4(c) and 4(d)). The segregated and porous surface property of S-SMSD explains the higher spreading of water, through easy penetration by capillaries in the pores presented on surface and thus formation of rapid fine microemulsion [10]. 

### 3.7. Solid-State Characterization of Solid SMEF

#### 3.7.1. Differential Scanning Calorimetry (DSC)

**Figure 5:**
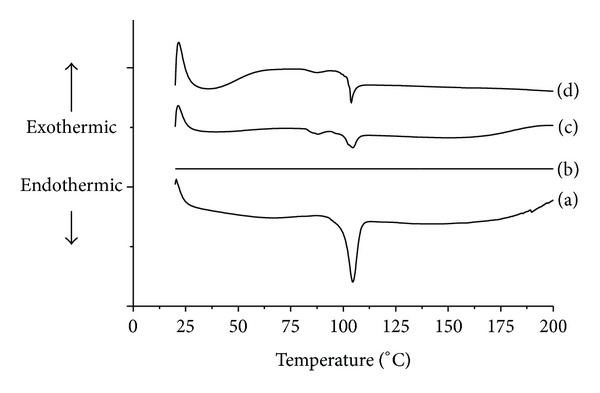
Differential scanning calorimetry of (a) valsartan, (b) Aerosil 200, (c) S-SMSD, and (d) S-SMFD.

The physical state of valsartan in pure form and in solid SMEF was investigated by DSC, and thermogram is presented in Figure 5. The thermogram of valsartan showed a sharp endothermic peak at about 108°C corresponding to its melting point. Aerosil 200 did not show any peak over the entire range of the tested temperatures (20–200°C). A broad with low melting enthalpy value is observed in S-SMSD formulation indicating that the drug is present in molecularly dissolved state. Moreover, the peak was prominent in SMFD and suggested that this method is not suitable for modifying the lattice arrangement of drug. The above finding suggested that the spray drying methods have prominent role in the changing in molecular structure of drug. 

#### 3.7.2. X-Ray Powder Diffraction (XRD)

**Figure 6:**
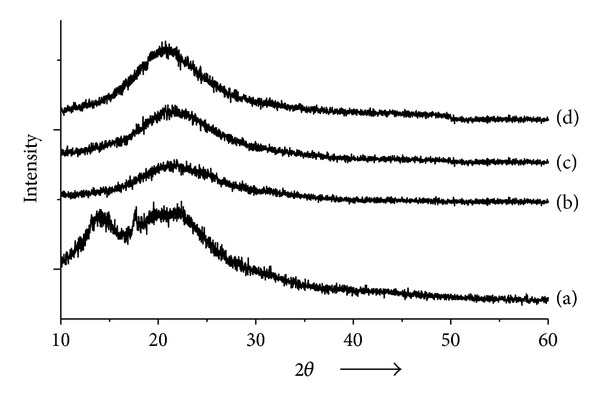
X-ray diffraction pattern of (a) Valsartan, (b) Aerosil 200, (c) S-SMSD, and (d) S-SMSD.

The internal physical state of valsartan in the solid SMEF and in pure form was further confirmed by XRD. The results of X-ray powder diffractograms of valsartan, Aerosil 200, S-SMSD, and S-SMFD are depicted in Figure 6. No characteristic peaks representing crystals were seen in the solid self micro-emulsifying formulation. The diffraction pattern indicated the changes in the crystalline nature of the drug in the formulations. The diffraction pattern of the valsartan exhibited its mixed nature, that is, amorphous and crystalline, indicated by two hump-shaped peaks. Relatively, there was less number of peaks observed in the diffraction pattern of solid SMEF. Presence of less instance peak in the S-SMSD as compared to S-SMFD can predict that a larger proportion of valsartan converted to amorphous form in the spray drying methods. The relative reduction in the diffraction intensities in the solid SMEF may be due to the change in the orientation of crystals or reduction in the types of crystals of valsartan. This change in diffraction pattern supported the conversion of crystalline to amorphous form.

### 3.8. Drug-Excipient Interaction Study

**Figure 7:**
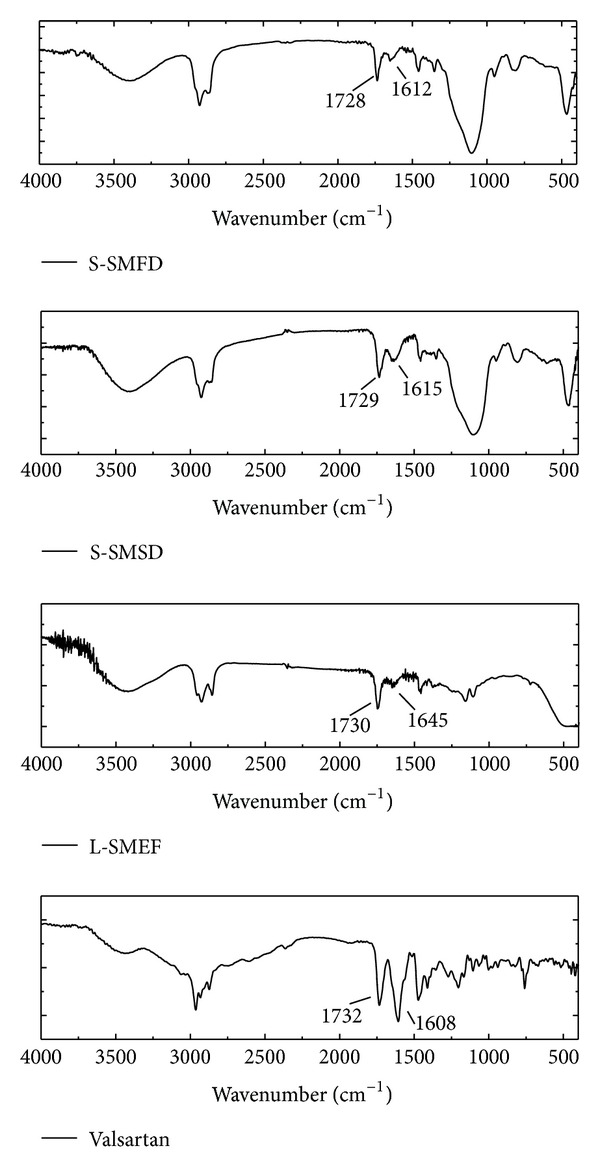
FTIR spectra of valsartan, L-SMEF, S-SMSD, and S-SMFD.

The I.R. spectrum of valsartan, liquid SMEF, and both solid SMEFs was taken, and the characteristic peaks were selected for drug lipids interaction study as shown in Figure 7. The valsartan alone showed two carbonyl absorption bands at 1732 cm^−1^ and 1608 cm^−1^, assigned to carbonyl-carbonyl and amide-carbonyl stretching, respectively. These bands are of diagnostic value to elucidate drug interaction with excipient. The carbonyl band of acid (1730 cm^−1^) and amide (1643 cm^−1^) stretching of valsartan in liquid SMEF indicated that there was no interaction between drug and lipids. There was no significance change in the carbonyl-carbonyl stretching in S-SMSD and S-SMFD. The shifting of wavenumber towards higher value (1647 cm^−1^) in S-SMSD and S-SMFD was within the range of amide group (1695–1600 cm^−1^). This peak shifting towards higher wavenumber with change in intensity suggested change in the environment of the carbonyl group associated with amide moiety. The slight shifting of absorption band for the carbonyl group of amide to a higher wavenumber can be attributed to the breakdown of the intermolecular hydrogen bonds associated with crystalline drug molecule and the formation of hydrogen bond of drug with excipient [26]. These illustrations and the similarity of IR spectra of valsartan and SMEF suggest absence of chemical drug-carrier interaction. 

### 3.9. *In Vitro *Dissolution Studies

**Figure 8:**
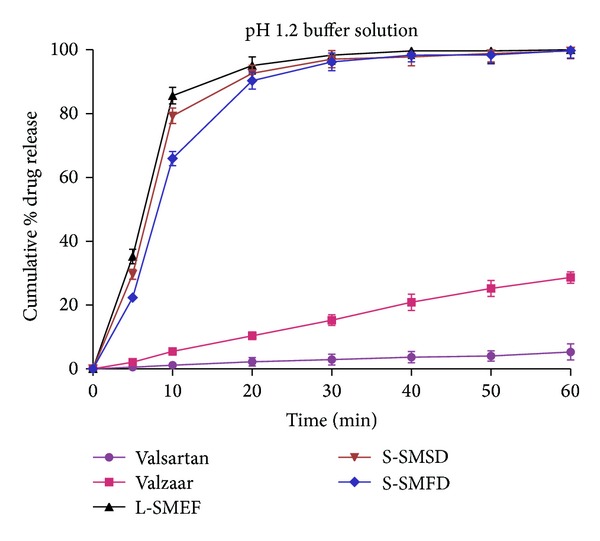
Dissolution of valsartan from different formulation in pH 1.2 buffer.

*In vitro* release experiments were conducted for the release of valsartan from the L-SMEF (F9), S-SMSD, S-SMFD, marketed capsule formulation (Valzaar 40 mg, Torrent India), and drug suspension. As valsartan is showing pH-dependent solubility behaviors, so it was necessary to evaluate the release of drug under different pH media for each formulation. As depicted in the graph (Figure 8), the average cumulative percent release of valsartan in pH 1.2 buffer solution from the L-SMEF, S-SMSD and S-SMFD within 20 minute was 95.06 ± 2.7%, 92.63 ± 2.53%, and 90.35 ± 2.65% respectively, whereas it was 2.23 ± 1.32% and 10.38 ± 1.15% for pure drug and valzaar, respectively. A significant increase in dissolution was observed with L-SMEF (F9), S-SMSD, and S-SMFD compared to valzaar and pure drug at gastric pH (pH 1.2). Initially, the slow release of drug from S-SMFD was due to unequal distribution of powder mass which took more time for emulsification and further dissolution. 

**Figure 9:**
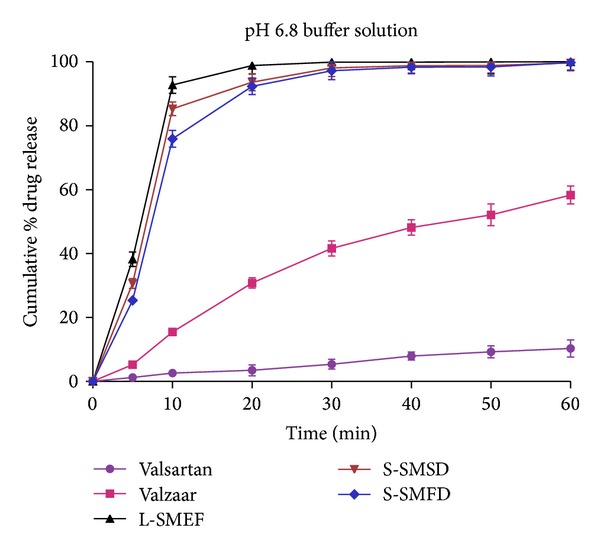
Dissolution of valsartan from different formulation in pH 6.8 buffer.

The release profile of drug was also evaluated in pH 6.8 phosphate buffer (Figure 9). As demonstrated by dissolution profile, more than 90% drug release was observed within 20 min from optimized liquid and solid SMEF, whereas it was 3.46 ± 1.74% and 30.78 ± 1.63% in pure drug and Valzaar, respectively. The L-SMEF (F9), S-SMSD, and S-SMFD, show pH-independent release of drug, whereas Valzaar and pure drug have pH-dependent release profile. The above finding clearly indicates the superiority of SMEF formulation over Valzaar in *in vitro* dissolution studies. 

### 3.10. Accelerated Stability Study

The results of stability studies of liquid and solid SMEF are shown in Table 5. There were no changes in their physical appearance in formulation. It was observed that the initial drug content and reconstituted microemulsion globule size of the samples analyzed at 0, 1, 2, and 3 months were similar, indicating that there were no significant changes in the appearance, globule size, and total drug content.

### 3.11. *In Vivo* Pharmacokinetic Study

**Figure 10:**
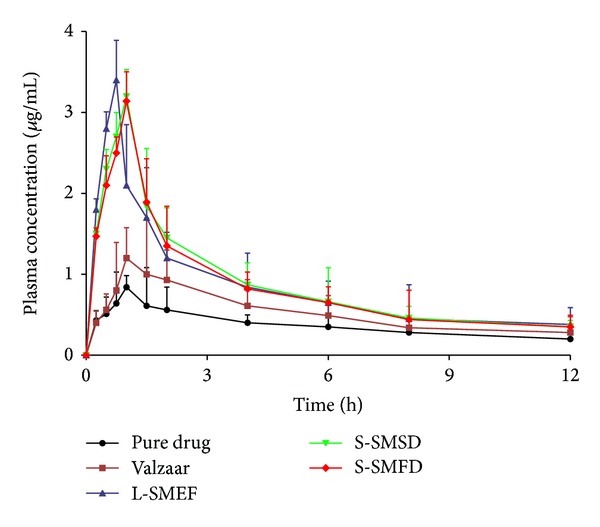
Plasma concentration-time profiles of the valsartan after oral administration of the pure drug, marketed formulation (Valzaar), and liquid and solid SMEF.

The pharmacokinetic parameter and relative bioavailability of liquid and solid SMEF were determined and compared with pure drug and marketed formulation. The mean plasma concentrations versus time profile of formulations and the pharmacokinetic parameters are shown in Figure 10 and Table 6, respectively. As data depicted, drug absorption from liquid and solid SMEF was significantly improved when compared to marketed formulation and pure drug. The liquid and solid SMEF indicated a significantly higher *C*_max⁡_ of the drug than valsartan powder or the commercial product (*P* < 0.05). Moreover, the AUC of the valsartan from the L-SMEF (F9), S-SMSD, and S-SMFD was approximately 1.5- and 3-fold higher than marketed formulation and pure drug respectively. However, the initial rate of absorption from solid SMEF (S-SMSD, S-SMFD) was slightly lower than liquid SMEF. It may be due to the initial slow dissolution of solid carriers. The plasma concentration time profile indicates the retention of microemulsion properties of liquid SMEF in solid dosage form with improved bioavailability. The result indicates enhancement in oral bioavailability of valsartan liquid and solid SMEF. It may be due to increase in dissolution rate of valsartan which makes higher concentration for systemic absorption. 

## 4. Conclusion 

In this present work, solid self-emulsifying formulation of valsartan was prepared by spray and freeze drying methods using Capmul MCM C8, Tween 80, and PEG 400 as carriers and Aerosil 200 as solid adsorbent. Both optimized solid SMEFs have globule size less than 200 nm and form instance microemulsion. The *in vitro* release studies depicted the pH-independent and fast release of drug from liquid and solid SMEFs whereas marketed formulation and pure drug showed pH-dependent characteristics. There was no significant difference in *in vitro* release of valsartan from S-SMSD and S-SMFD, and this was also demonstrated in *in vivo* bioavailability studies. The relative bioavailability of solid SMEF was approximately 1.5- to 3.0- fold higher than marketed formulation and pure drug. The XRD, DSC, and FTIR results revealed that the drug is present in molecular state. Both spray and freeze drying methods have been found to be potential for the solidification of liquid SMEF. However, the S-SMSD exhibited superior characteristics as compared to S-SMFD in terms of spherical porous particles, uniformity in size, better flow properties, and rapid processing. The improved efficacy of the solid SMEF was established by the preclinical studies as compared to marketed formulation; however the final clinical studies are required for the further evaluation and effective therapeutic application. 

## Figures and Tables

**Table 1:** Composition of different batches of various liquid SMEFs.

Batch	Valsartan (% w/w)	Lipid oil (Capmul MCM C8) (% w/w)	Surfactant (Tween 80) and cosurfactant (PEG 400) ratio		
			(1 : 1) (% w/w)	(2 : 1) (% w/w)	(3 : 1) (% w/w)
F1	20	40	40	—	—
F2	20	32	48	—	—
F3	20	24	56	—	—
F4	20	16	64	—	—
F5	20	8	72	—	—
F6	20	40	—	40	—
F7	20	32	—	48	—
F8	20	24	—	56	—
F9	20	16	—	64	—
F10	20	8	—	72	—
F11	20	40	—	—	40
F12	20	32	—	—	48
F13	20	24	—	—	56
F14	20	16	—	—	64
F15	20	8	—	—	72

**Table 2:** Evaluation parameters of liquid SMEF bathes.

Batch	Emulsification efficiency	Freeze thawing	Globule size (nm) (mean ± SD)	Polydispersity index (mean ± SD)	Zeta potential (mV) (mean ± SD)
F1	D	Unstable	1152 ± 124	0.404 ± 0.021	2.2 ± 0.031
F2	C	Unstable	813 ± 69	0.346 ± 0.012	2.9 ± 0.045
F3	C	Unstable	700 ± 57	0.346 ± 0.025	2.7 ± 0.034
F4	B	Stable	306 ± 30	0.321 ± 0.032	3.2 ± 0.052
F5	A	Stable	189 ± 21	0.280 ± 0.052	3.4 ± 0.032
F6	C	Unstable	350 ± 35	0.234 ± 0.024	2.5 ± 0.030
F7	C	Unstable	249 ± 32	0.356 ± 0.035	3.0 ± 0.042
F8	B	Stable	206 ± 28	0.333 ± 0.031	3.5 ± 0.024
F9	A	Stable	127 ± 16	0.233 ± 0.032	4.6 ± 0.023
F10	A	Unstable	105 ± 14	0.691 ± 0.035	3.9 ± 0.052
F11	D	Unstable	1238 ± 237	0.500 ± 0.042	1.6 ± 0.063
F12	D	Unstable	1016 ± 210	0.426 ± 0.014	1.6 ± 0.024
F13	C	Unstable	989 ± 189	0.621 ± 0.054	2.0 ± 0.063
F14	B	Stable	403 ± 26	0.262 ± 0.023	2.1 ± 0.022
F15	A	Stable	197 ± 18	0.307 ± 0.031	3.0 ± 0.043

**Table 3:** Particles size of reconstituted liquid and solid SMEF.

Formulation	Particles size (nm)	Polydispersity index	Drug content (%)
L-SMEF	127 ± 16	0.233 ± 0.032	99.32 ± 2.13
S-SMSD	133 ± 28	0.244 ± 0.052	97.59 ± 3.62
S-SMFD	160 ± 37	0.479 ± 0.027	99.14 ± 1.56

**Table 4:** Powder flow characteristics of solid SMEF.

Formulation	Angle of repose (*θ*)	Bulk density		Carr's index (%)	Hausner ratio
		Tapped density (mg/cc)	Fluff density (mg/cc)		
S-SMSD	30 ± 2	0.606 ± 0.042	0.488 ± 0.017	19.50 ± 1.3	1.242 ± 0.21
S-SMFD	38 ± 4	0.373 ± 0.031	0.278 ± 0.025	25.54 ± 1.2	1.34 ± 0.14

**Table 5:** Stability studies of liquid and solid SMEF.

Formulation	Evaluation parameters	Observations (months)			
		0	1	2	3
L-SMEF (F9)	Physical appearance	Pale yellow clear liquid	Pale yellow clear liquid	Pale yellow clear liquid	Pale yellow clear liquid
	Globule size (nm)	127 ± 16	130 ± 24	132 ± 13	133 ± 32
	Total drug content	100.0 ± 1.4	99.51 ± 0.9	99.50 ± 1.0	99.48 ± 1.2

S-SMSD	Physical appearance	White powder	White powder	White powder	White powder
	Globule size	133 ± 28	135 ± 42	136 ± 24	136 ± 14
	Total drug content	100.0 ± 0.9	99.29 ± 1.2	99.09 ± 1.1	99.02 ± 0.9

S-SMFD	Physical appearance	White powder	White powder	White powder	White powder
	Globule size	160 ± 37	162 ± 23	163 ± 21	165 ± 34
	Total drug content	100.0 ± 0.9	99.10 ± 0.8	99.08 ± 1.3	99.03 ± 1.6

**Table 6:** Pharmacokinetic parameters of valsartan after oral administration of the pure powder, marketed formulation (Valzaar), and liquid and solid SMEF.

Parameters	Pure drug	Valzaar	L-SMEF	S-SMSD	S-SMFD
*C*_max⁡_ (*μ*g/mL)	0.83 ± 0.14	1.20 ± 0.23	3.40 ± 0.89	3.20 ± 1.23	3.14 ± 1.13
*T*_max⁡_ (h)	1.00 ± 0.21	1.10 ± 0.42	0.75 ± 0.24	1.02 ± 0.53	1.05 ± 0.46
*t*_1/2_ (h)	7.83 ± 1.23	5.13 ± 1.47	5.94 ± 1.25	6.06 ± 2.63	5.22 ± 2.35
Kel (h^−1^)	0.088 ± 0.03	0.13 ± 0.07	0.11 ± 0.04	0.11 ± 0.06	0.13 ± 0.03
AUC *t*_0_–*t*_12_ (*μ*g h/mL)	3.45 ± 0.95	6.33 ± 1.75	10.23 ± 2.57	10.69 ± 2.14	10.28 ± 1.95
AUC *t*_0_–*t*_∞_ (*μ*g h/mL)	6.71 ± 1.34	8.41 ± 2.42	13.48 ± 4.53	13.76 ± 3.73	12.92 ± 2.47
*f*_1_	—	—	2.9	3.0	2.98
*f*_2_	—	—	1.6	1.68	1.62

*f*_1_: Relative bioavailability compared to pure drug.

*f*_2_: Relative bioavailability compared to marketed formulation.

Each value is represented in mean ± SD. *n* = 3.
